# Assessing the Usability of Six Data Entry Mobile Interfaces for Caregivers: A Randomized Trial

**DOI:** 10.2196/humanfactors.4093

**Published:** 2015-12-15

**Authors:** Frederic Ehrler, Guy Haller, Evelyne Sarrey, Magali Walesa, Rolf Wipfli, Christian Lovis

**Affiliations:** ^1^Division of Medical Information SciencesDepartment of medical imaging and medical information sciencesUniversity Hospitals of GenevaGenevaSwitzerland; ^2^Division of AnaesthesiologyUniversity Hospitals of GenevaGenevaSwitzerland; ^3^Division of Clinical EpidemiologyUniversity Hospitals of GenevaGenevaSwitzerland; ^4^Department of Epidemiology and Preventive MedicineMonash UniversityMelbourneAustralia; ^5^Direction of NursingUniversity Hospitals of GenevaGenevaSwitzerland; ^6^School of MedicineUniversity of GenevaGenevaSwitzerland; ^7^Faculty of medicineUniversity of GenevaGenevaSwitzerland

**Keywords:** data collection, mobile applications, computers, handheld, user-computer interface, vital signs, patient safety

## Abstract

**Background:**

There is an increased demand in hospitals for tools, such as dedicated mobile device apps, that enable the recording of clinical information in an electronic format at the patient’s bedside. Although the human-machine interface design on mobile devices strongly influences the accuracy and effectiveness of data recording, there is still a lack of evidence as to which interface design offers the best guarantee for ease of use and quality of recording. Therefore, interfaces need to be assessed both for usability and reliability because recording errors can seriously impact the overall level of quality of the data and affect the care provided.

**Objective:**

In this randomized crossover trial, we formally compared 6 handheld device interfaces for both speed of data entry and accuracy of recorded information. Three types of numerical data commonly recorded at the patient’s bedside were used to evaluate the interfaces.

**Methods:**

In total, 150 health care professionals from the University Hospitals of Geneva volunteered to record a series of randomly generated data on each of the 6 interfaces provided on a smartphone. The interfaces were presented in a randomized order as part of fully automated data entry scenarios. During the data entry process, accuracy and effectiveness were automatically recorded by the software.

**Results:**

Various types of errors occurred, which ranged from 0.7% for the most reliable design to 18.5% for the least reliable one. The length of time needed for data recording ranged from 2.81 sec to 14.68 sec, depending on the interface. The numeric keyboard interface delivered the best performance for pulse data entry with a mean time of 3.08 sec (SD 0.06) and an accuracy of 99.3%.

**Conclusions:**

Our study highlights the critical impact the choice of an interface can have on the quality of recorded data. Selecting an interface should be driven less by the needs of specific end-user groups or the necessity to facilitate the developer’s task (eg, by opting for default solutions provided by commercial platforms) than by the level of speed and accuracy an interface can provide for recording information. An important effort must be made to properly validate mobile device interfaces intended for use in the clinical setting. In this regard, our study identified the numeric keyboard, among the proposed designs, as the most accurate interface for entering specific numerical values. This is an important step toward providing clearer guidelines on which interface to choose for the appropriate use of handheld device interfaces in the health care setting.

## Introduction

Electronic data collection and recording in the health care setting is performed increasingly at the patient’s bedside. Data (eg, medical prescriptions, medical summary reports, or daily recordings of body temperature, respiratory or cardiac frequency) can easily be collected on portable computers. Such devices have the advantage of being easy to store, manipulate, and use in emergency departments, outpatient clinics, or other crowded areas. Among the portable devices on offer, tablets and smartphones are becoming increasingly popular due to their handiness and resemblance to traditional paper-and-pencil data collection interfaces [1-3]. They also offer the advantage of providing apps designed especially for handheld devices, such as drug dosage calculators, electronic pharmacopeias, textbooks, or medical literature databases [1,4-7].

In the health care setting, many usability problems contribute to medical errors [8], of which those related to data entry are a major source. The quality of recorded information is of utmost importance because the life of a patient can easily be put at risk by the improper recording of a drug dosage or the incorrect labeling of a physiological or biological value [9-11]. Accuracy in the process of data recording can be significantly influenced by the design of an interface or by factors related to the type of data to be recorded, such as a number’s length, type, magnitude or frequency, and even font appearance [12]. This has already been demonstrated for specific entry devices, such as infusion pumps [12-14], and in the context of medical prescriptions [8]. The limited size and tactile interaction paradigm of handheld devices compared to traditional laptops has further emphasized the risk of increased errors in data entry [3].

The influence that specific characteristics of handheld devices can have on human-machine interactions has already been studied [15-27]. Earlier works investigated the impact of limited display size on users’ performances for tasks such as browsing, information retrieval, readability, or target selection [15-19]. These studies showed that the size of a mobile screen has no major impact on the user’s comprehension of the information displayed. However, a correlation was found between the ease of reading and the size of the screen. In this context, Duchnicky and Kolers [20] found that it takes users up to 25% longer to read a given text on a small-width display than on a regular desktop screen width. This was confirmed by another study by Resiel and Shneiderman [21] who reported that reading on a 22-line display compared with a 60-line display resulted in a 15% decrease in the speed of reading. The same is true for information retrieval; studies have indicated that information retrieval tasks are harder to perform on small screen devices [22]. Moreover, users are more likely to perform incorrect choices when selecting from possible links and waste more time carrying out additional scrolling type activities [23,24]. Screen size also influences the quality of information input. Most studies assessing users’ performance have confirmed that data input accuracy can be impacted by keyboard size or character setting, but also by other factors, such as a user’s finger size [25-27]. Generally, a familiar disposition of the display and large keyboards improve user performance.

All these tend to demonstrate that it is crucial to take into account screen size specificity in the design of mobile device interfaces. Therefore, we assessed 6 tactile interfaces representing many of the common interfaces used on tablet PC and smartphones as interfaces built based on user requirements.

## Methods

In order to identify the most suitable interface for the effective and reliable recording of numerical data on handheld devices, we performed a crossover randomized trial assessing 6 handheld interfaces designed according to 6 paradigms ranging from commercially available solutions to experimental designs. Each participant had to record several vital numerical signs on each interface. The interfaces were provided automatically in a random order.

### Participants

A sample most representative of health care professionals likely to collect, record, or use clinical data as part of their daily activity was recruited within the University Hospitals of Geneva, Switzerland. Recruitment was carried out following approval of the project by the “Commission cantonale d'éthique de la recherche,” the University hospitals’ human research and ethics committee. Previously published research on similar research questions were able to demonstrate significant differences between data entry interfaces with 30 participants involved [13]. However, because we were able to recruit among a large number of caregivers and had no prerequisites as to participants’ characteristics, we relied on a convenience sampling approach. In total, 150 hospital workers selected across 5 categories of professionals were recruited: nurses, assistant nurses, midwives, physicians, and administrative staff. Participation was on a voluntary basis and there was no exclusion criterion.

### Instruments

**Figure 1:**
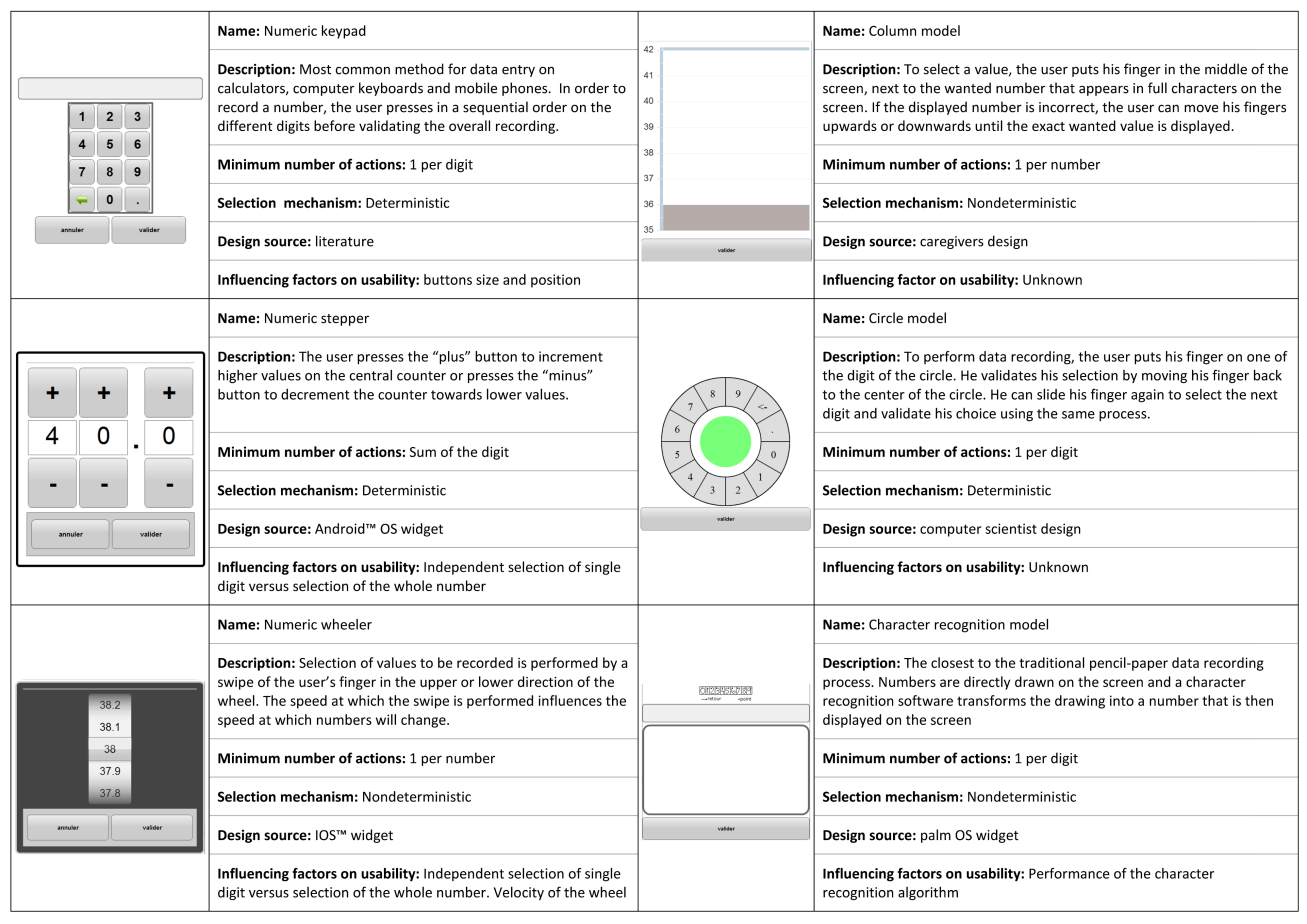
Description of the six data entry interfaces.

To test the 6 interfaces, we used a common commercially available smartphone, the Samsung Galaxy Note, which has a 5.3-inch screen with a resolution of 1280×800 pixels. This type of smartphone is representative of contemporary smartphones. It is also characterized by a high level of flexibility, which reduces to the minimum the design constraints set by human-machine interfaces. The 6 interfaces that were tested were designed to represent the most complete range of options man-machine computer interfaces can offer (Figure 1). Four of the interfaces were chosen among existing interface design frameworks. The numeric keypad interface was chosen because it is recognized as being a being very effective at reducing the number of entry errors [13]. The stepper and wheel interfaces were chosen because they are commonly available on 2 major commercial smartphone operating systems, Android and iOS. Finally, the character recognition interface was chosen because it was among the first interfaces proposed for touchscreen devices, such as the PalmPilot. The last two options were developed by two distinct expert committees based on end-user requirements for recording interfaces. The first committee included caregivers who designed a dynamic chart (named “column”) aimed at facilitating the identification of vital sign trends. The second committee included computer scientists at the hospital who developed a fast data entry system (the “circle”) built on an interaction principle similar to the SwiftKey keyboard. The interfaces were implemented by the research team and installed on all devices provided to participants. Experimental designs were previously extensively tested by volunteers to ensure usability of the recording interfaces.

### Study Procedure and Data Collection

The trial was designed to minimize interaction biases. Based on a set of possible problems identified in a pilot phase, standardized procedures were defined so that any problem (eg, a system crash) would by default always be handled in a similar way. Each session of the trial took place in a controlled environment where participants could perform the test without any interruption or other disturbance. Before beginning the trial, each participant was shortly briefed on the study purpose and overall procedure. Participants were also informed that there was no time constraint for entering the displayed numbers. Each participant was then provided with a smartphone. Because the study procedure and instructions were available on each device and the entire study process was regulated by a computer program, there was no need for further interaction with the research team. Although the experiment took place in a controlled environment, participants were asked to use only one hand to hold the device to simulate the real-life bedside recording procedure as closely as possible (see Multimedia Appendix 1 for the CONSORT checklist filled for this study).

**Figure 2:**
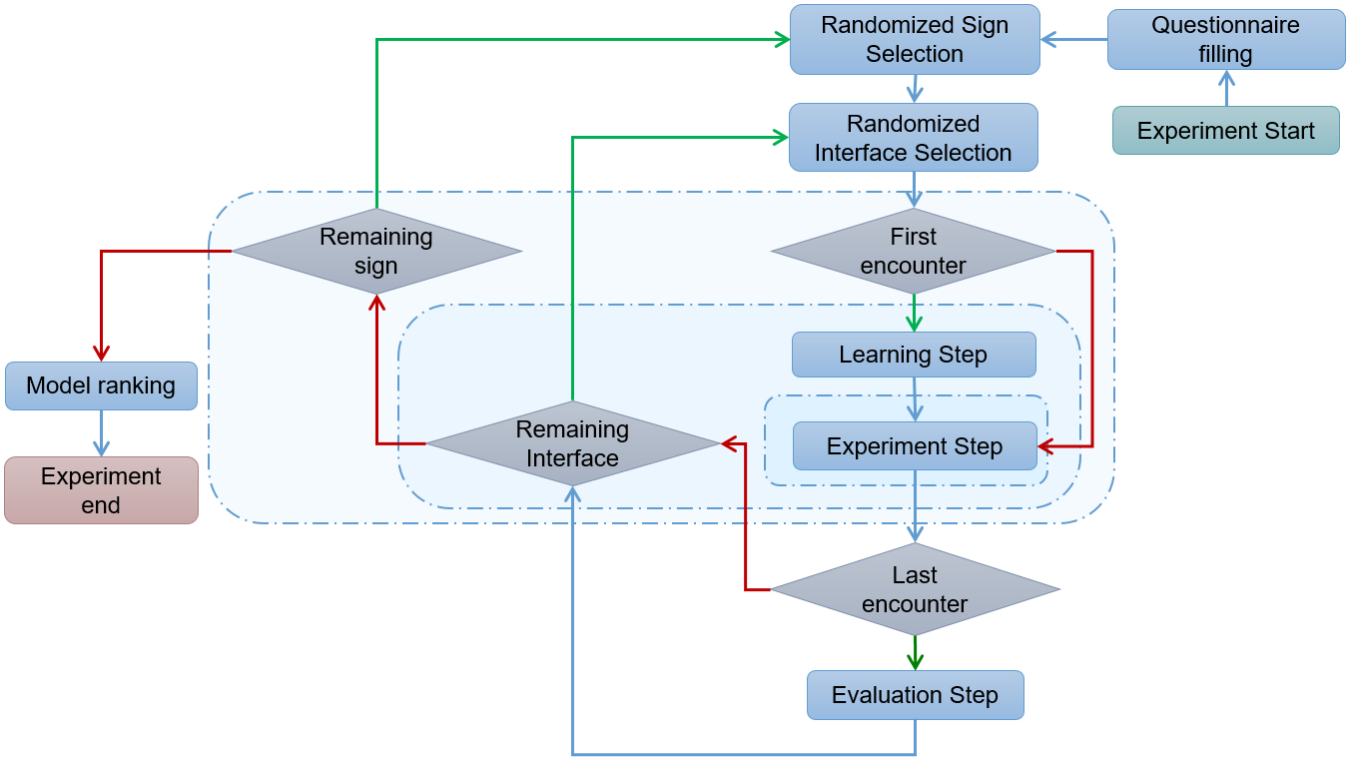
Data recording process.

Participants were first asked to read the instructions of the experiment that were displayed on the smartphone. A short questionnaire then popped up on the screen asking for details on participants’ demographics and computer literacy. Following these steps, the actual trial phase began. Each participant was requested to enter data on all 6 interfaces. The sequence in which interfaces came up was automatically computed and randomly defined for each participant. The procedure was the same for each interface: participants were first able to practice the procedure before entering the data to be recorded. For each interface design, participants were asked to enter 3 types of physiological measures (body temperature, respiratory rate, pulse rate) that were displayed at the top of the screen. We chose these 3 vital signs from among the most frequently collected signs during patient follow-up that could be described using a single numerical value. For this reason, measurements such as blood pressure (composed of 2 values) were discarded as were other types of data, such as dates or time, which we considered would require other types of dedicated entry interfaces. When entering a number, participants could correct it as many times as they wanted before validating it. The computer program randomly generated the type of physiological data to be recorded and a value to be entered. During the training period, participants had to record 2 physiological values with the interface displayed on the screen. Users could not skip this step and had to continue the selection process until they succeeded. This is the only time users were allowed to request external assistance if they did not understand how to use the interface. Once the 2 physiological values were successfully recorded, the testing process started and participants were asked to record 3 random values for each possible interface-sign combination. Data for pulse, respiratory rate, and body temperature were given a predefined range of recordable values (Table 1).

**Table 1:** Range of recordable values.

Sign	Range	Decimal	Number of possible values
Pulse	30-170	No	140
Body temperature	36-41	Yes	50
Respiratory rate	3-20	No	17

The task was repeated until 3 values were recorded for each of the 3 physiological signs in each of the 6 interfaces. Altogether, this totaled 54 data entries for each participant.

### Measured Variables

All variables needed for assessing the performance of each participant were recorded automatically on the smartphone. Among the variables that were measured were the number of actions performed by the participant, the number of corrections made, and the time until data entry validation. Data could be exported in a comma separated value file. Accuracy was measured by comparing the values participants were requested to enter as they appeared on the top of the smartphone’s screen with the ones actually entered by each participant. Success or failure outcomes were then summed and reported as a proportion of the maximum possible score for each category of data entry. Effectiveness was assessed by measuring the time used to record each of the values.

### Analysis

Descriptive summary statistics of continuous variables included means (SD), or medians with ranges, depending on their distribution. Continuous variables were compared using the paired Student *t* test or the Wilcoxon signed rank test if not normally distributed. For categorical variables, we used frequencies and proportions. Participants’ satisfaction was assessed using the Friedman 2-way ANOVA test. After adjusting for age and familiarity of use of computers or smartphones, possible associations between the data entry time for each type of variable and the type of interface used or the sequence of data entry were examined using multilevel linear models. The same was done to evaluate the number of errors and corrections associated with each interface, this time using multilevel mixed logistic models. To obtain a normal distribution of the dependent variable, we used the log of speed of data entry. Interfaces were nested within the type of data entry, which were themselves nested within the coder.

A *P* value <.05 was considered statistically significant. We performed all analyses using the statistical package Stata version 12.

## Results

Only 4 participants failed to complete the test due to technical problems or professional emergency causing them to leave the study before its completion. These incomplete records were removed from the study analysis. Demographics for the remaining 146 participants could be summarized as follows: mean age of participants was 43.6 years (SD 10.2) with two-thirds (64.4%, 94/146) younger than age 46 years. Most of participants enrolled in the study (92.5%, 135/146) were right-handed and nearly all (95.9%, 140/146) possessed a personal computer, although only 61.0% (89/146) already owned a smartphone. Most (75.3%, 110/146) were caregivers; the remaining 24.7% (36/146) were recruited among administrative personnel or computer scientists classified as “other participants.” Study participant characteristics are detailed in Table 2.

The time needed to enter values with the different interfaces differed depending on the type of vital sign recorded (Table 3). In most cases, it took less time to enter pulse than body temperature values, and even less time to enter respiratory rates (except for the wheel mode). The results provided in Table 3 show that the speed of data entry was also influenced by the type of interface used. Differences were statistically significant (*P*<.001). The fastest interface for data entry was the numeric keyboard, with a mean entry speed ranging from 2.81 (SD 0.06) to 4.34 (SD 0.08) seconds depending on the type of variable recorded. This was significantly quicker than the stepper (mean 4.31, SD 0.11 seconds to mean 7.23, SD 0.36 seconds), the wheeler (mean 5.13, SD 0.14 seconds to mean 8.35, SD 0.21 seconds), and the circle (mean 4.45, SD 0.01 seconds to mean 9.38, SD 0.28 seconds) models. Finally, the less efficient interfaces were the column (mean 5.25, SD 0.16 seconds to mean 10.49, SD 0.45 seconds) and character recognition (mean 6.32, SD 0.24 seconds to mean 14.68, SD 0.64 seconds) models.

Table 4 shows data entry accuracy for the different interfaces. The numeric keyboard was the most accurate interface (96.3%-99.3% accuracy). It was followed by the stepper (97.9%-98.4% accuracy) and the wheel (95.4%-98.6% accuracy). Other interfaces were associated with more data recording errors. The other interfaces yielded levels of accuracy ranging from 93.2% to 94.3% for the column model, 88.6% and 96.1% for the circle, and 81.5% to 86.8% for the character recognition interface.

Among the tested interfaces, the numeric keyboard achieved the highest level of accuracy for recording pulse data. When looking at the mean scores of all 3 measures, the overall performance of the numeric keyboard was comparable to that of the stepper and wheel models. With regard to speed of data entry, the numeric keyboard scored the highest, with results at least 1.5 times faster than all other interfaces regardless of the type of variable recorded. Interfaces built based on a participative design, such as the circle or column, were associated with additional errors. Character recognition was found to be the most inaccurate and slowest interface for clinical data recording (Table 5).

## Discussion

These study results can be explained by several factors. First, the selection mechanism used to enter values into the system was the most influential factor for determining speed of data entry. With a deterministic selection mechanism, such as the keyboard, the actions performed by users are transformed unambiguously into the associated outcome. This differs from a nondeterministic selection mechanism, such as character recognition, where there is no guarantee that the action of the user is transformed into the desired outcome. The interfaces with such nondeterministic selection mechanisms require some level of expertise and training to perform accurate actions that can be transcribed by the system into the desired outcome. The number “1” could, for instance, be at misinterpreted as a “7” and therefore require additional time to be corrected. The second parameter influencing the speed of data entry is the number of actions required to record it. In this regard, the stepper is not as fast as the keyboard because it requires more actions to enter a given numerical value.

An analysis of the error rate enabled us to classify the interfaces into 2 main categories: those yielding a level of accuracy greater than 95% (numeric keyboard, stepper, and wheel) and those yielding levels less than 95% (character recognition, circle, and columns). Interfaces offering no immediate feedback and those where it was difficult to modify recordings (character recognition, wheel) were associated with a significantly increased risk of recording errors.

**Table 2:** Participant characteristics (N=146).

Participant characteristics		n (%)
**Age (years)**		
	22-34	42 (28.8)
	35-45	52 (35.6)
	≥46	52 (35.6)
**Gender**		
	Female	96 (65.7)
	Male	50 (34.3)
**Hand preference**		
	Left-handed	11 (7.5)
	Right-handed	135 (92.5)
**Profession**		
	Assistant nurse	4 (2.7)
	Nurse	76 (52.2)
	Administrative	19 (13.0)
	Midwife	11 (7.5)
	Physician	19 (13.0)
	Other	17 (11.6)
**Has a computer**		
	No	6 (4.1)
	Yes	140 (95.9)
**Has a smartphone**		
	No	57 (39.0)
	Yes	89 (61.0)
**Frequency of vital sign recording**		
	Never	45 (30.8)
	Monthly	12 (8.2)
	Every day	89 (61.0)

**Table 3:** Mean time to enter 3 variables for each interface-sign combination.

Data type	Interface entry time (sec), mean (SD)						*P*
	Character recognition	Circle	Columns	Numeric keyboard	Stepper	Wheel	
Pulse	10.50 (0.44)	6.27 (0.14)	10.06 (0.34)	3.08 (0.06)	6.53 (0.36)	8.35 (0.21)	<.001
Body temperature	14.68 (0.64)	9.38 (0.28)	10.49 (0.45)	4.34 (0.08)	7.23 (0·18)	7.22 (0.19)	<.001
Respiratory rate	6.32 (0.24)	4.45 (0.01)	5.25 (0.16)	2.81 (0.06)	4.31 (0.11)	5.13 (0.14)	<.001

**Table 4:** Correct data entries for the 3 variables on each of the 6 interfaces.

Data type	Correct data entries, n (%)						*P*
	Character recognition	Circle	Columns	Numeric keyboard	Stepper	Wheel	
Pulse	372 (84.9)	421 (96.1)	413 (94.3)	435 (99.3)	431 (98.4)	432 (98.6)	<.001
Body temperature	357 (81.5)	388 (88.6)	412 (94.1)	425 (97.0)	431 (98.4)	427 (97.5)	<.001
Respiratory rate	380 (86.8)	413 (94.3)	408 (93.2)	422 (96.3)	429 (97.9)	418 (95.4)	<.001

**Table 5:** Data entry speed and accuracy for different interface characteristics.

Interface characteristics	Mean time (sec)	Mean accuracy, %	Minimum number of actions measured	Selection	Design source
Numeric keyboard	3.41	97.5	1 per digit	Deterministic	Literature
Stepper	6.02	98.2	Sum of the digits	Deterministic	Android OS
Circle	6.7	93	1 per digit	Deterministic	Computer scientists
Wheeler	6.9	97.2	1 per number	Nondeterministic	iOS
Columns	8·6	93.9	1 per number	Nondeterministic	Caregivers
Character recognition	10.5	84.4	1 per digit	Nondeterministic	Palm OS

Our results are in line with other research findings [13,28,29]. A study comparing 5 number entry interfaces for an infusion pump [13] showed lower error rates and speed of data entry when entering values on a number pad rather than a stepper. In a study evaluating data recording accuracy of a keyboard for electronic health recording systems compared with handwritten medical paper records, authors found that 25.6% of vital signs had one or more errors when documented on paper medical records compared to 14.9% errors when documented in an electronic format [28]. This confirms the improved accuracy of a keyboard compared to handwriting, as used in character recognition interfaces. Another study by Wager et al [29] compared error rates for 4 different types of entry devices (paper, computer on wheel, tablet, and direct feed from the monitoring system). Although no details were provided about the interfaces used, the error rate associated with a keyboard on a tablet was 5.6%, on average, similar to our study findings.

There are several limitations to our study. The first is the lack of exact knowledge about the prior level of familiarity users had for each of the 6 interfaces. However, one can reasonably assume that the novel interfaces implemented for the study were unknown to all users, whereas numeric keyboard and character recognition interfaces were probably familiar to computer and mobile phone users. Our study sample was mostly composed of the latter (see Table 2). In order to minimize this bias, we included a training session for each interface in our protocol. It is unclear whether this was sufficient to ensure a similar level of mastery for all categories of interfaces, but this limited the risk of variability due to insufficient training rather than to a true difference between interfaces [30,31].

A second limitation is the unknown influence of the original parameters set into the system on study outcomes [32,33]. Indeed, there are many factors that can influence the data entry process. For instance, the size of the keyboard buttons is known to influence a user’s performance during data entry [27,34]. On infusion pumps, such factors can generate up to 28 different implementations of the data entry interface [13]. In their study, Cauchi et al [35] confirmed that these parameters not only influence data entry performance, but also the severity/degree of recording errors. Likewise, the character recognition algorithm can facilitate or complicate user inputs [36]. Therefore, if our results show clear and statistically sound differences in interface usability and accuracy, other studies analyzing the same interfaces but using different settings may show different results.

Third, despite explicitly instructing participants to act as they would normally during the trial session, while at the same time forbidding observers to intervene after the testing phase, it cannot be excluded that external observers may have had an influence on participants’ behavior. For example, participants may have improved or modified an aspect of their behavior in response to the context in which they were acting rather than in response to the type of device used. This is known as the “Hawthorne effect” [37].

Fourth, the experimental setting cannot be considered as an ecologically valid one because the experiment took place in a controlled environment. Although we attempted to minimize this limitation by asking users to carry the device in only one hand, the fact that the numbers to enter were provided directly on the smartphone did not reflect real-life situations, where numbers are more likely to be read from another source. We chose this method of data delivery to exclude errors that might otherwise arise when transcribing data from one place to another [38].

Fifth, the purely quantitative approach adopted in our study cannot provide explanations as to why variations exist across different interfaces and what the implications are for the design and evaluation of mobile data entry tools. To clearly answer these questions, a more qualitative methodology needs to be adopted, such as the one used by Kushniruk et al [8] in their analysis of the relationship between usability problems and prescription errors in handheld applications.

Finally, it is also worth pointing out that evaluating failure based on a binary measure does not make it possible to evaluate the type and potential severity of an error. Indeed, studies such as those carried out by Wiseman et al [39] or Oladimeji et al [13,40] reported up to 7 classes of errors. Due to this limitation, our results do not highlight the fact that interfaces that enable each digit to be chosen independently are much more likely to create errors of higher magnitude and usually critical in a clinical context [12,41,42].

Although there is a marked increase in use of handheld devices in the health care environment, not all interfaces are adapted to specific constraints, such as smaller, interactive tactile screens. The possibility of recording clinical information on mobile devices opens the door to many questions. Interfaces set on these devices are usually complex to use. In his study, Howard [43] showed that this complexity could be explained by 4 reasons: intricacy, equivalence, omniscience, and commitment. To account for these different aspects, the design of a new interface should evaluate each parameter separately for effectiveness and accuracy before combining parameters into a more complex design. Moreover, the identification of the most appropriate handheld user interface to record numerical data in an electronic format is only one of the many aspects that need to be investigated. Further aspects, such as the manipulation of text data or the recording of graphical data, need to be analyzed also.

Recording information in real time on handheld devices at the patient’s bedside is increasingly becoming the standard of care in many health care settings. These devices offer improved portability and flexibility compared to desktop computers. The ease and accuracy with which data can be recorded in such settings will determine the choice of the most appropriate human-machine interface. Although a lot of work has been done on physical keyboards by engineers and ergonomists to improve the reliability and efficiency of data recording, a lot of work still has to be done for handheld devices. Our study shows that among the interface designs we selected, the keyboard reached the highest level of speed and accuracy when recording pulse data. The stepper and wheeler interfaces demonstrated similar accuracy, whereas the numeric keyboard remained by far the quickest interface for tactile interaction. Whether this conclusion will remain valid when using other interface parameters remains to be tested; however, the need for more in-depth evaluation of novel interfaces has clearly been demonstrated.

Although some interface designs may, at first sight, appear promising, only formal and rigorous assessments in randomized trials will enable the identification of the most accurate and usable interfaces for data recording in the clinical setting. As the new generation of handheld devices progressively replaces traditional computers, future developments to find the most appropriate human-machine interface should not only be based on designer or user committee inputs, but also on advice from other fields, such as experts in ergonomics.

## Multimedia Appendix 1

CONSORT checklist.
